# Concerns and Goals of Women with Isolated Mesh-Associated Pain Syndrome Accessing a Quaternary Mesh Referral Service: A Thematic Analysis

**DOI:** 10.1007/s00192-025-06323-7

**Published:** 2025-10-21

**Authors:** Hawra Badri, Lucy Dwyer, Claire Serridge, Kelechi Ajoku, Karen Ward, Richard Edmondson, Fiona Reid

**Affiliations:** 1 Warrell Unit, Saint Mary’s Hospital, Manchester University NHS Foundation Trust, Manchester Academic Health Science Centre, Hathersage Road, Manchester, M13 9WL UK; 2 Manchester Academic Health Science Centre (MAHSC), Manchester, UK; 3 Division of Developmental Biology & Medicine, School of Medical Sciences, Faculty of Biology, Medicine and Health, University of Manchester, Manchester, UK; 4 Division of Nursing, Midwifery and Social Work, School of Health Sciences, Faculty of Biology, Medicine and Health, University of Manchester, Oxford Road, Manchester, M13 9PL UK; 5 Burnley General Hospital, East Lancashire NHS Foundation Trust, Blackburn, UK; 6 Division of Cancer Sciences, School of Medical Sciences, Faculty of Biology, Medicine and Health, The University of Manchester, Manchester, UK; 7 Department of Gynaecological Surgery, Manchester University NHS Foundation Trust, Manchester, UK; 8 Division of Pharmacy and Optometry, School of Health Sciences, Faculty of Biology, Medicine and Health, University of Manchester, Manchester, UK

**Keywords:** Mesh complications, Pelvic pain, Stress urinary incontinence

## Abstract

**Introduction and Hypothesis:**

Isolated mesh-associated pain syndrome (I-MAPS) is the commonest reason why women access mesh-complication services. Qualitative work exploring expectations of women with I-MAPS is limited. We aimed to explore the concerns and goals of women with I-MAPS who accessed care at a quaternary-level mesh service to ensure that services are designed to meet their needs.

**Methods:**

A total of 280 women with I-MAPS related to a single continence device, were invited to provide free-text comments on concerns and goals related to their mesh complication using the Electronic Patient Assessment Questionnaire (e-PAQ). Of 280 participants, 203 completed the e-PAQ (response proportion 73%) and 179 (response proportion 64%) provided comments. Thematic analysis was performed based on the methodology proposed by Braun and Clarke.

**Results:**

Thirty-eight codes were developed, and 109 sub-codes. These defined eight core themes. Concerns regarding pain accounted for 22% of comments (106 out of 489). Concerns about pelvic floor symptoms featured heavily in comments. Symptom resolution was desired by most; however, a proportion requested symptom reduction. Mesh-removal surgery was a common goal pursued; however, a proportion wished information about the safety and future threat of mesh devices.

**Conclusions:**

Patients with I-MAPS appear to have concerns unrelated to pain including pelvic floor dysfunction and these may be the primary motivation for accessing mesh services. Although mesh removal was a motivation for many, it was not requested by all. This highlights the importance of services offering individualised and holistic care through multidisciplinary team involvement.

## Introduction

Worldwide, over 3 million polypropylene mesh devices have been implanted for the management of stress urinary incontinence (SUI). Mesh continence devices were widely considered to be both a safe and effective alternative to traditional continence surgeries, associated with high failure rates and morbidity [1]. As a result, mesh continence procedures became the gold-standard management for SUI. This resulted in a proliferation of the use of mesh for continence surgery [2] and paved the way for the development of mesh devices for pelvic organ prolapse (POP).

Within a decade of widespread adoption of mesh devices concerns developed owing to adverse events experienced [3–5]. Scrutiny of mesh devices instigated predominantly by the public and affected women in the UK, were instrumental in influencing a cascade of litigations, regulatory alerts and commissioning of an independent parliamentary review culminating in eventual suspension of all vaginally placed mesh, which continues to be enforced. Complications reported by affected women included mesh infection, vaginal exposure, perforation into viscera and pain. Pain has been reported to be the most common complication associated with mesh devices, and recognising the prevalence and impact of mesh-related pain, the European Association of Urology introduced the term mesh-associated pain syndrome (MAPS) [6]. We have previously identified that MAPS in the absence of other mesh complications is the most widely reported complication experienced by mesh-affected women in our service [7], which we have termed isolated mesh-associated pain syndrome (I-MAPS).

The voice of affected women featured heavily in Baroness Cumberledge’s 2021 review [8] in outlining the harm experienced from mesh devices and missed opportunities to prevent it. Increased emphasis on involvement and incorporation of perspectives of service users in health care research was amongst the recommendations stipulated by the review, which were resonated in the 2022 Women’s health strategy for England [9]. To date research into mesh complications remains limited, with quantitative research being the focus of most research efforts. To our knowledge, no studies in publication have explored the experiences of women with I-MAPS and reasons why this patient cohort access care at mesh-complication services.

The aim of this study is to understand the concerns, goals and motivations of women with I-MAPS related to a single continence mesh device who access care at our quaternary mesh-complication service. The findings of this study are intended to support quality improvement efforts and to provide insights for future areas of qualitative research required.

## Materials and Methods

All women referred to our quaternary mesh-complication service between 26 January 2018 and 19 April 2024 with I-MAPS related to a single continence device were asked to complete a questionnaire. Patients not reporting I-MAPS or who had other mesh-related complications were excluded. The questionnaire was the Electronic Patient Assessment Questionnaire (e-PAQ), which is a validated questionnaire and was sent to women on referral to the service. Patient responses were collected retrospectively. As well as multiple-choice responses, patients were invited to provide free-text comments on concerns related to their condition and goals for their treatment at the mesh service. Patients were asked:

“Concerning the issues that currently concern you the most, what do you hope to achieve from any help, advice or treatment?”

This approach allowed patients to relay their experiences unrestricted by closed lines of questioning. The e-PAQ assessment was undertaken as part of routine clinical care at the mesh service and hence regulatory approval was not required for this study.

Patients were invited to complete the e-PAQ assessment on referral to the mesh service. Non-responders were prompted to complete the assessment at mesh clinic appointments. A good level of literacy in the English language was required to complete the questionnaire.

The thematic analysis was performed based on the methodology proposed by Braun and Clarke [10]. Themes were developed using the six stages of reflexive thematic analysis:

1. Familiarisation with the data: This was performed independently by HB and LD. HB is a doctoral candidate with a clinical background in women’s health and who worked closely within the mesh-complication service. LD is a nurse researcher with expertise in qualitative research and a clinical background in urogynaecology. However, she was not affiliated with the mesh service and was thus able to provide an objective, unbiased interpretation of the data, free from pre-conceived ideas. The data were analysed by a third reviewer, CS, who is an experienced psychosexual psychotherapist working within the mesh service who provided feedback on the accuracy of data interpretation.
2. Coding of the data: This was performed separately by the authors and involved summarising data into short phrases or words. Codes were discussed by HB and LD to ensure that the codes accurately reflected the data, and that any nuance was not lost.
3. Generation of initial themes: This was performed using an inductive approach, whereby data were organised and labelled to allow the identification of patterns and relationships between the data sets, which were then organised into central themes.
4. Development and review of themes: Following review, consolidation and assessment of codes, the authors worked collaboratively to develop core themes that were believed to accurately represent information provided in the patient comments. Themes were created to reflect patterns and trends identified from the responses.
5. Refinement of themes: Themes were reviewed and analysed against the available codes and following further discussion between the authors, core themes were agreed.
6. Writing up of results: This report was developed following the interpretation of the themes as described by Braun and Clarke.

## Results

**Fig. 1:**
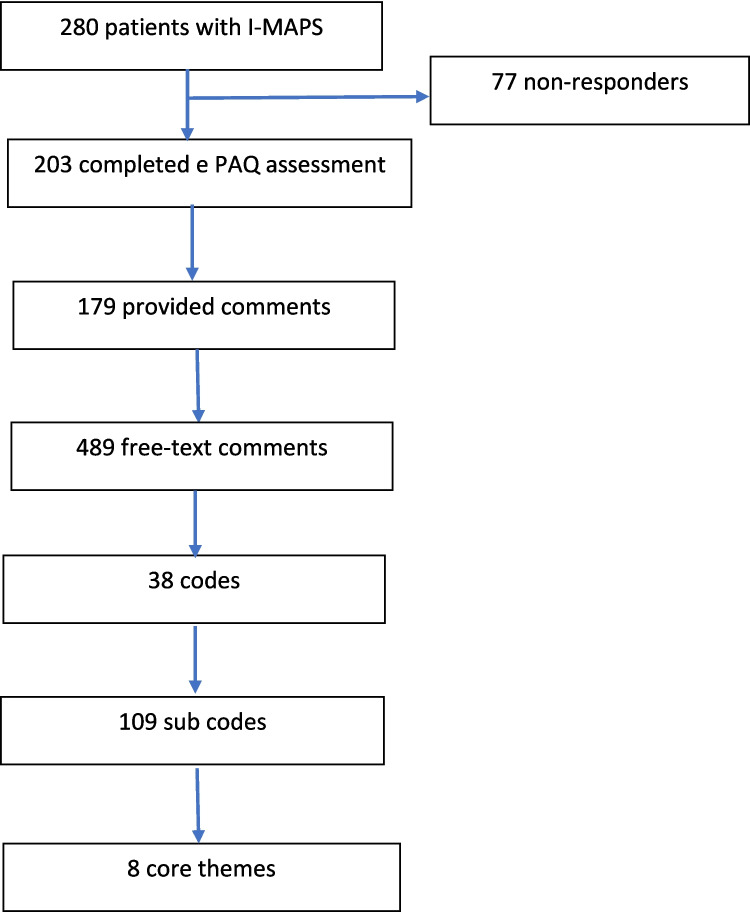
Flowchart representing the process of theme development

Two hundred and eighty women with I-MAPS related to a single continence mesh device were managed in the mesh-complication service over 5 years. Two hundred and three completed the e-PAQ assessment (response proportion 73%), of whom 179 provided free-text comments (response proportion 64%; Fig. 1).

The demographics of the patient groups are provided in Table 1. The demographics of women who completed the e-PAQ questionnaire were overall non-significantly different from non-responders. Women with I-MAPS were predominantly post-menopausal and obese, with over half of patients (52%; 146 out of 280) reporting additional pain-inducing medical conditions including fibromyalgia and osteoarthritis. The median Index of Multiple Deprivation (IMD) based on postcode for patients with I-MAPS was 5, indicating that deprivation within this cohort was within the middle of the spectrum. IMD ranks neighbourhoods in England from 1 (most deprived area) to 10 (least deprived area) based on indices including educational attainment and income [11].

**Table 1:** Demographics of the group of women with isolated mesh-associated pain syndrome and comparison between Electronic Patient Assessment Questionnaire responders and non-responders

Demographic data	Responders (*N* = 203)	Non-responders (*n* = 77)	*p* value
Age, median (range) [IQR]	60 (33–94) [57–60]	61 (42–53) [58–61]	0.99
BMI, median (range) [IQR]	30 (19–47) [26–34]	29 (22–39) [26–34]	0.91
Index of Multiple Deprivation, median (range) [IQR]	5 (1–10) [3–8]	6 (1–10) [2–8]	0.76
Smoking, *n* (%)	22 (11)	4 (5)	0.17
Pain conditions, *n* (%)	112 (55)	34 (44)	0.11
Autoimmune conditions, *n* (%)	21 (10)	4 (5)	0.24
Mood conditions, *n* (%)	34 (17)	11 (14)	0.72
Diabetes, *n* (%)	22 (11)	1 (1)	0.007

**Fig. 2:**
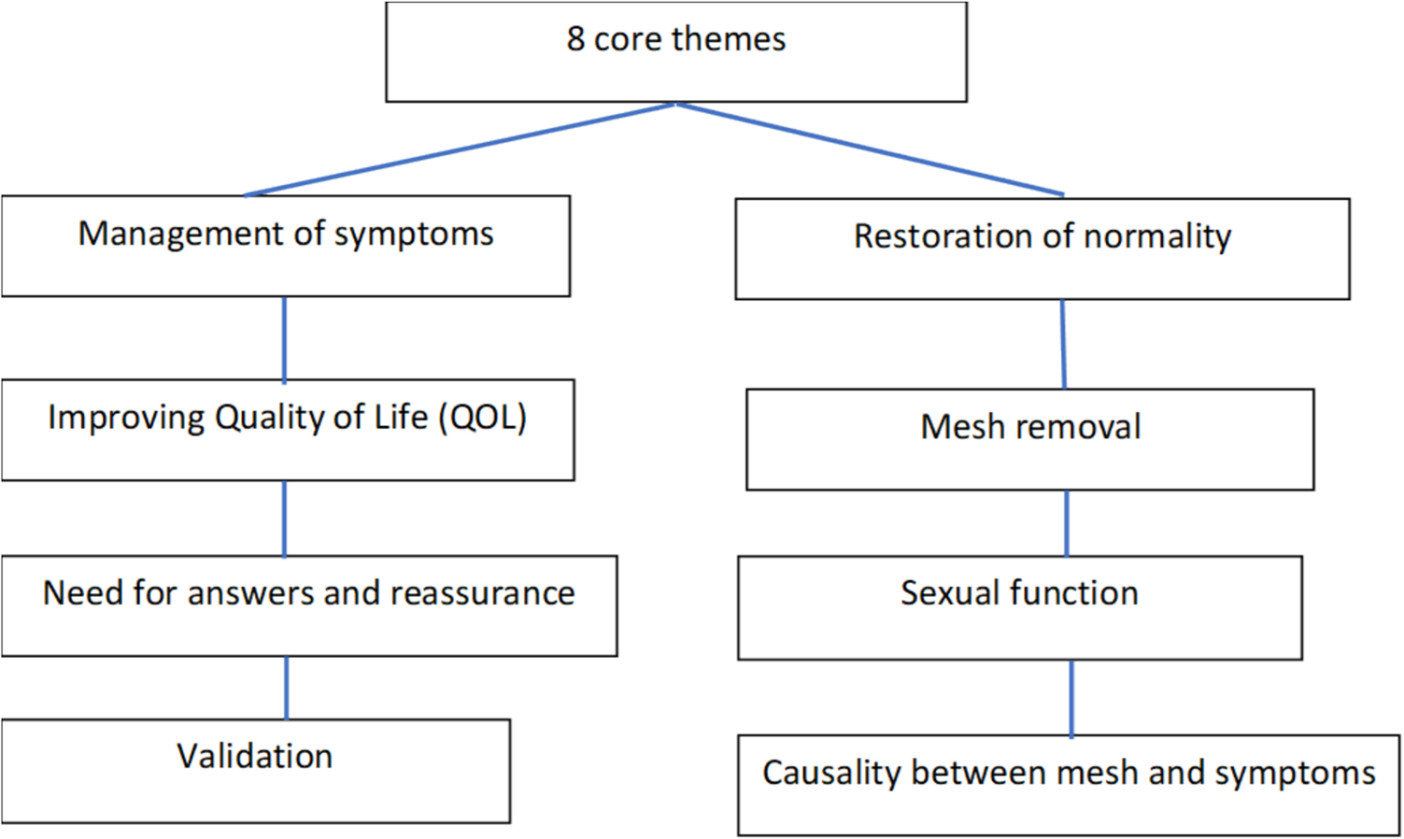
The eight core themes

In total, 38 codes were developed, further defined by 109 sub-codes. Eight core themes were defined that encompassed the codes, and these are illustrated in Fig. 2.

### Management of Symptoms

The impact of pain on women and their lives featured heavily in responses. Pain intensity was described using words such as “excruciating”, “extreme” and “agony”. Comments included a desire for “freedom”, “control”, “eradication” and “relief” from pain.

Although I-MAPS was the primary reason for referral to the mesh service in this study group, concerns related to pain featured in only 22% of comments (106 out of 489). Women described concerns related to several pelvic floor symptoms unrelated to pain, including prolapse, urinary incontinence, bowel symptoms, bleeding and recurrent urinary tract infections (rUTIs). There appeared to be a spectrum of expectations regarding symptoms, with some women desiring improvement:

“To reduce toilet trips and frequency.”

“Decrease in urinary infections”.

“Better control over bowels”.

Ohers pursued complete resolution of their symptoms:

“A cure for my incontinence”.

“To be infection free”.

“To stop leakage from anus”.

Many women expressed anguish at how long these symptoms had been occurring and the effect that they had on their self-view.

Responses included reference to symptoms that were “constant”, “continual”, “uncontainable” and some reported suffering for years.

“I have had TVT for 10 years. Constant pain”.

Women reported feeling “unclean” owing to urinary

 and faecal incontinence, reported frustration with the persistent odour of urine and how this had left them feeling “self-conscious”, “embarrassed” and “lonely”.

### Improving Quality of Life

This core theme incorporated women’s perceptions of how their current symptoms had a detrimental effect on their quality of life (QOL). Pain was described as a barrier to mobilisation, which had an impact on women’s functional ability and enjoyment of life; therefore, many described a wish to improve this as a treatment goal:

“To be pain free and able to walk”.

“Less pain in groin and legs when walking and doing general activities”.

“To wake up and look forward to having pain-free day without anxiety about pain and discomfort”.

Similarly, urinary and faecal incontinence were reported by some as having an impact on their ability to leave their homes and resulted in feelings of anxiety and a sense of isolation. Worries about needing to “toilet-map” when outdoors featured in several comments. These concerns were not specifically described as being related to their mesh devices.

“Being able to go out and not worry about finding a toilet to empty my bowels and not feeling scared to go out”.

“To feel confident to go out and not wet myself or have urge incontinence constantly”.

“To be able to go out and not worry about messing myself”.

Many responses included general and non-specific statements of wishing to feel “better”, healthy” and to be able to function as markers of improvement and of good quality of life that were goals of accessing care at the mesh service:

“Just to get on with life”.

“I need to feel better. I am so tired of being poorly”.

“Just able to enjoy life and not feel ill all the time”.

“Better overall quality of life, which includes work home and social life”.

### Need for Answers and Reassurance

A common theme identified from the responses was the importance of receiving answers and explanations regarding symptoms experienced:

“I would like to have my questions answered about the TVT mesh and for the consultant to advise me”.

Women expressed concerns and anxieties about their future health if mesh were to remain within their body and wished to receive advice on this. Choices regarding treatment options was valued and considered empowering. Although mesh removal was requested by many women, a proportion of women instead desired information and reassurance regarding the integrity, position and condition of their mesh device:

“To understand that the tape is still intact and not interfering with back or bowels”.

“Check status of mesh to ensure it’s not eroding”.

“I would like advice on the state of the vaginal TVT mesh. How will this impact further on my health.”

“Advice and support on the position of the mesh, options to leave or removal”.

“To have confidence that pain will not get worse or increase in future”.

### Validation

This theme demonstrates many women’s feelings that they had not been listened to or believed in the past regarding their symptoms, as well as a desire to have their symptoms and concerns acknowledged or recognised by health care providers. Women expressed frustration with having concerns dismissed. Some responses exemplify dissatisfaction with previous health care encounters and signify a loss of trust, including the perception of having been lied to or causing them to question the reality of their symptoms, through “gaslighting”.

“I want to be listened to, honest truthful diagnosis as I’ve been messed about for last 5 years”.

“Explanation of misdiagnosis!”.

“Someone to take me seriously”.

“That I’m being listened to and believed”.

### Restoration of Normality

This theme describes a treatment goal reported by women to regain control and normality in their lives through treatment of their symptoms. Many women perceived their current conditions to be abnormal and felt unable to resume or progress in their lives until their symptoms were treated. Several comments featured a wish to “live again”.

“Ability to start living my life again”.

“Give me a plan forwards to full healing to be able to live life fully again”

“How to get my life back on track after years of hell!”

“I would like to have a normal life without worrying about leakage and a good night’s sleep”.

### Mesh Removal

A desire for mesh removal featured several times in the responses. Mesh removal was pursued by women to remedy current symptoms thought to be secondary to the mesh device and to prevent future complications.

“I hope to have this TVT tape removed because it has ruined my life and would like to be pain free”.

“To have my mesh removed to prevent further deterioration in health”.

Mesh devices were associated with injury or damage to their body which was cited by some as a motivation to request mesh removal:

“To be mesh free before it does any more damage”.

Women were insightful regarding the consequences of mesh removal including the possibility of developing recurrent stress urinary incontinence and wished for their mesh device to be substituted with a “safer” alternative.

“I really hope they are able to remove it and put something safer in its place”.

### Sexual Function

The perceived impact of mesh on women’s sex life was a common finding in the responses. Women detailed pain and incontinence as barriers to intimacy and described breakdown in relationships as a result. Comments describe feelings of embarrassment associated with incontinence and shame regarding perceptions of an abnormal body image:

“Discomfort in the vagina and no intimacy with husband since 2011 impacting on my marriage”.

“Happy sex life and a tight vagina etc”.

“Not be so embarrassed with my partner having to make an excuse if I am having a bad bladder day”.

“Have a relationship and a partner, so not so lonely”.

“Move onto a new relationship and have a normal life and sex life free of pain and embarrassment”

“Vaginal shame gone and regain our love life”.

One woman perceived difficulties with having a sexual relationship with their spouse as threatening to their identity as a woman:

“To be able to feel like a proper woman and have a proper marriage, and sexual life. Not done for years”.

### Causality Between Mesh and Symptoms

The relationship between symptoms experienced, development of conditions and presence of mesh within the body were questioned by many women. Women attributed symptoms such as pain, bleeding, infection, allergies and autoimmune disorders to mesh devices:

“Discussion about whether mesh is causing other health problems”.

“Are conditions I have that have been attributed to other conditions in fact reactions to my TVT mesh?”.

“I would like to be guided through the issue of my MESH causing my health to deteriorate”.

“To identify if my transvaginal tape is causing these issues. Operation in 2009. Now multiples issues”.

“I have bleeding from my vagina when opening my bowels possibly caused by the TOT I had done.”

## Discussion

This qualitative exploration incorporating the concerns and goals of 179 women with I-MAPS who accessed a quaternary-level mesh-complication service, provides an insight into the experiences and management pursued by this patient cohort. We aimed to explore their expectations to better understand treatment goals for mesh-affected women. This will enable us to ensure that care provision within our mesh service meets women’s needs. Our findings indicate that despite I-MAPS being the principal reason for referral, management of conditions precipitated by pelvic floor dysfunction appear to be the focus of many women’s concerns and desires for their treatment at the mesh service. These conditions included prolapse, rUTI and urinary and faecal incontinence. These conditions are prevalent within the post- menopausal population and pose diagnostic and management challenges. Although concerns regarding pain featured less within responses, chronic pain posed a devastating and far-reaching impact within multiple domains, including physical, psychological, social and sexual. Although mesh removal was requested by many women, others instead sought an improvement in their QOL, or informational empowerment. Equally, women wished their experiences to be heard, validated and affirmed. These findings highlight the importance of health care professionals listening, shared decision making, high-quality information provision and avoidance of paternalistic health care. The findings also indicate a disintegration of trust between mesh-affected women and health care providers and a request from women for honesty, autonomy and choice.

We identified that despite the media attention surrounding mesh complications, many women with I-MAPS were accessing the mesh service for symptoms unrelated to their mesh devices. Pelvic floor dysfunction posed a considerable impact on women’s QOL in keeping with findings within the literature [12, 13]. These findings were echoed by another qualitative study undertaken between 2018 and 2020 when media coverage of mesh complications was at its height that identified a similar patient emphasis on pelvic floor health and related QOL [14]. This highlights the importance of effective communication and involvement of patients in health care, a view endorsed by the General Medical Council, which encourage doctors to avoid assumptions regarding patient needs and expectations of care, but rather to “find out what matters to patients” [15]. This resonated further in a study that explored surgeons’ reasoning for continuing mesh implantation, which revealed surgeons’ pre-occupation with restoration of anatomy rather than patient satisfaction [16]. For women with I-MAPS accessing our service, treatment goals took many forms, from management of co-existing pelvic floor dysfunction, improvement of QOL, information and advice, and for some, mesh removal surgery.

We uncovered the destructive nature of pain and pelvic floor symptoms, which were far reaching and all encompassing, affecting all domains of life. Responses encapsulated the physical, social and emotional impact, including feelings of shame, humiliation and loss of dignity, as well as the erosion of confidence, relationships and for one woman her sense of female identity. Williams et al. [17] corroborates these findings in their thematic synthesis of qualitative studies of women with pain and other pelvic mesh complications, where they reference two core themes of “broken body” and “broken mind” to attest to the widespread adverse effects of pain and mesh complications. This was echoed by Toye et al. [18], who describes mesh complications “stealing” individuals’ dignity and sense of self. A recurring theme in qualitative work involving patients who have sustained iatrogenic harm is fury and anguish at harm being “done to” them [17, 19] and whether it was preventable. This provides another potential reason for distrust of health care professionals.

Responses highlighted a distrustful and strained patient–doctor relationship, with reference to being dismissed and not believed. The Cumberledge report [8] presents similar experiences of women facing unempathetic attitudes from health care providers, and normalisation of symptoms. Williams et al. describes a feeling of “double betrayal” in mesh-affected women who endorsed feelings of anger at being misled about the success and likelihood of complications of the original mesh surgery and then being disbelieved and denied care for the post-surgical complications. Patients with pain felt especially more vulnerable to dismissal and perceived their symptoms to be overshadowed by more visible mesh complications [17], further exacerbating feelings of “gaslighting”. Toye et al. reports patient accounts of being made to feel “neurotic” or “hysterical” and that their symptoms were overamplified [18]. Gender bias in medicine is well documented and has led to minimisation and disregard of symptoms reported by women. One qualitative systematic review [20] reports on the harm of discounting patient experiences, which further compounds the psychological harm of mesh complications.

Women’s wish for validation and understanding of the relationship between symptoms and their mesh device further signifies the erosion of trust. Encountering dismissive medical professionals, many women found solidarity in online communities, termed “knowledge in communitas” [17]. These provided them with not only support but also alternative sources of knowledge on their mesh complications and empowered many to challenge complication denial. Knowledge acquired through these communities reinforced beliefs of health care professionals being fallible. Toye et al. proposes the need to “re-negotiate trust” by treating patients as “embodied wholes” rather than a pathology and being receptive to alternative perspectives [18]. Our findings can therefore contribute to an improvement in care provision for mesh-affected women by highlighting the complex and varied goals and expectations of women attending a mesh service and that assumptions should not be made regarding this. Within our service, these findings have been used to ensure that patient expectations are thoroughly explored and that the diversity of management choices available are clearly outlined. Multi-format patient decision aids are routinely used in conjunction with patient consultations to support informed decision making, ensure transmission of evidence-based information and reduce decisional conflict.

### Strengths

The strengths of this study include the inductive approach using an unrestricted line of questioning through free-text comments without categorised or pre-defined responses. To our knowledge this is the first qualitative study involving only patients with I-MAPS in publication. Our mesh service is amongst the largest in the UK, our study size of 179 is likely to be one of the largest single-centre cohorts of women with I-MAPS and therefore findings are more likely to be representative of this patient cohort.

### Limitations

The study is limited by the single-centre design and inherent methodological limitations of response and recall bias. Qualitative research has been criticised for being biased by introducing the subconscious bias and perceptions of the researchers. This was especially relevant as two of the authors (HB and CS) worked closely within the mesh service. We have attempted to mitigate for this through inclusion of a researcher independent of the mesh service (LD) and through independent development of codes and sub-codes before discussing and finalising themes. The design of the study requiring written response is also a limitation in that patients with lower levels of literacy were excluded. Use of verbal responses through interviews would be more inclusive and allow better representation of patient views of the population served by the mesh service. This study did not elicit any information regarding patient engagement in litigation for mesh complications. We recognise the possibility of this influencing patient-reported outcomes.

### Interpretation and Future Work

This study revealed three needs of this patient cohort when accessing mesh-complication services. Patients with I-MAPS often experience other co-existing pelvic floor disorders that may have a larger impact on QOL. Clinicians must ensure that attention is not focussed solely on mesh complications and that assumptions are not made about management expectations. Services should ensure tailored and individualised care that addresses individual patient needs.

Second, this work revealed that many women with I-MAPS experience devastating and far-reaching consequences of their symptoms. Although many desire surgery, a proportion seek knowledge, explanation and reassurance. This supports the importance of providing patients with access to multi-disciplinary teams capable of offering holistic care including psychotherapy, psychosexual services and physiotherapy. Participants clearly valued information and choice. This supports the need for high-quality and evidence-based patient information resources and emphasis on shared decision-making health care models within mesh services.

Third, we identified the broken trust between doctor and patient. Acknowledgement of harm suffered by mesh devices and full disclosure regarding the risks and success rate of proposed treatment options are essential to rebuilding lost trust.

Future qualitative research is required to inform the efficacy of treatment options including surgical and non-surgical treatments of I-MAPS. This work is intended to inspire further qualitative work exploring patient motivations for requesting surgical and non-surgical therapies in larger, multi-centre patient cohorts.

## Conclusion

Women with I-MAPS report concerns unrelated to pain, including pelvic floor dysfunction, which may be the primary motivation for accessing care at mesh-complication services. These symptoms are profound and far-reaching for women. Although mesh-removal surgery was pursued by many, this was not requested by all. Improved QOL, knowledge, choice and validation were also valued. This highlights the need for mesh services to be patient centred and offer individualised, multi-modal and holistic care through involvement of multidisciplinary teams.

## Data Availability

The data that support the findings of this study are not openly available due to reasons of sensitivity and are available from the corresponding author upon reasonable request.
